# North Carolina Dental Association

**Published:** 1867-05

**Authors:** 


*North Carolina Dental Association.*—It is gratifying to note that our friends in North Carolina are endeavoring, by well directed efforts, to elevate the standard of the profession in their State.

We are also pleased to see that the graduates of the Baltimore College of Dental Surgery are at the head of this movement No less than six out of the nine officers of the Association being graduates of the Baltimore College. They propose to have a law passed by the Legislature which requires every one who commences dental practice in the State after a specified time, to be a graduate of a dental college, and that those who have been in practice prior to the time named, be required to submit to an examination by a regularly appointed board of the State Dental Society.

